# Flexion–extension cervical MRI imaging in pediatric patients with achondroplasia unsupervised by neurosurgery or radiology, is it safe?

**DOI:** 10.1007/s00381-025-06777-6

**Published:** 2025-02-25

**Authors:** Aseel Masarwy, Christopher Watterson, Alexander Tuchman, Moise Danielpour

**Affiliations:** 1 Neurosurgery Department, Cedars Sinai Medical Center, Los Angeles, CA USA; 2 Radiology Department, Cedars Sinai Medical Center, Los Angeles, CA USA

**Keywords:** Achondroplasia, Cervical spine, Flexion–extension MRI, Safety

## Abstract

**Background and purpose:**

Achondroplasia, a common form of skeletal dysplasia, can be associated with cervical spine compression and cerebrospinal fluid (CSF) flow compromise, potentially leading to neurological complications. Accurate assessment of the cervical spine is essential for identifying children at increased risk of neurological injury. However, concerns regarding the safety of dynamic MRI under anesthesia in young children have limited its use. This study evaluates the safety of dynamic MRI under anesthesia in pediatric patients with achondroplasia, utilizing the largest dataset reported to date.

**Materials and methods:**

In this retrospective study, we reviewed the medical records of 81 patients with achondroplasia who underwent a total of 124 flexion–extension MRIs under anesthesia. All imaging procedures were performed by MR technologists and anesthesiologists without direct supervision by a neurosurgeon or radiologist. Data reviewed included anesthesia type, neurological examinations by a senior pediatric neurosurgeon before and after imaging, surgical intervention, follow-up MRIs, and the presence of CSF obstruction at the craniocervical junction.

**Results:**

A total of 81 patient charts were reviewed (mean age, 2.03 ± 2 years; age range, 1 month to 6 years). Of the 124 flexion–extension MRIs, 113 were performed under general anesthesia, and 11 under sedation alone. Foramen magnum stenosis with CSF flow compromise was documented in 38 cases (46%). No adverse events, neurological deficits, or anesthesia-related complications were documented. Neurological examinations conducted by the senior author, a pediatric neurosurgeon, before and after imaging remained stable across all cases.

**Conclusion:**

Flexion–extension MRI did not result in adverse outcomes in this cohort of pediatric patients with achondroplasia. While these findings support the dynamic MRI’s safety in appropriate settings, further studies are needed to validate these results and explore its broader application.

## Introduction

Achondroplasia is the most common form of skeletal dysplasia, caused by an activating mutation in the fibroblast growth factor receptor 3 (FGFR3) gene [1]. This mutation results in abnormalities in cartilaginous bone growth, which underlie all clinical manifestations and complications of this condition. These abnormalities may arise directly or from disproportionate growth of cartilage relative to adjacent structures [2]. The hallmark clinical features of achondroplasia include shortening of the extremities, macrocephaly, frontal bossing, and joint hypermobility [2]. Premature closure of the synchondrosis and fusion of ossification centers [3], particularly at the cranial base, can lead to stenosis of the foramina at the skull base, including the foramen magnum. As a result, patients with achondroplasia are at risk for craniocervical compression, spinal stenosis, hydrocephalus, and even sudden death [4].

Symptoms of craniocervical compression often include abnormal neurological findings such as hyperreflexia, clonus, abnormal tone, central sleep apnea, neck hyperextension, motor developmental delay, gait disturbance, paresthesia of the extremities, and weakness [5]. Additionally, these symptoms are sometimes accompanied by signal abnormalities visible on MRI [6]. Treatment for symptomatic patients typically involves craniocervical junction decompression, which is especially important for children at the highest risk of sudden death [7]. Criteria for decompression have been previously described in large surgical series [7, 8]. However, given the risks and potential complications of surgery, it is critical to accurately identify high-risk patients who could benefit most from surgical intervention [7].

Several imaging modalities are used to evaluate the craniocervical junction, but dynamic magnetic resonance imaging (MRI) provides excellent visualization and enables the assessment of cord compression [9]. Additionally, dynamic flexion–extension MRI offers additional diagnostic insights by revealing compression that may only become evident on dynamic studies [5]. Because spinal canal compromise may occur in infants and young children with achondroplasia, these studies frequently require sedation. However, many institutes remain skeptical about the use of cervical flexion–extension MRI, due to concerns over potential harm, including transient cord compression leading to apnea or cord contusion, versus the diagnostic benefits. Consequently, the use of this imaging modality remains limited to a few centers with significant experience and comfort in managing such patients.

There are only a few studies that address the safety of dynamic MRI in children with skeletal dysplasia. Mackenzie et al. [10] described a retrospective series of 31 patients with skeletal dysplasia, including 6 with achondroplasia, who underwent dynamic MRI under sedation or anesthesia. The study showed that the procedure could be safely performed with adequate supervision and provided essential guidance for medical and surgical decision-making. Similarly, Mukherjee et al. [5] reported on dynamic craniocervical compression in 29 patients with achondroplasia, highlighting the utility of flexion–extension MRI as a diagnostic tool for symptomatic patients, particularly when neutral cervical spine MRI findings were unremarkable. While these studies provide useful insights, our review of the literature found no safety assessment in a large cohort of pediatric patients with achondroplasia undergoing flexion–extension MRI, highlighting the need for more research in this area.

In this single-center retrospective study, we present the largest dataset reported to date of pediatric patients with achondroplasia undergoing dynamic MRI under anesthesia without neurosurgical supervision. This study aims to assess the safety of this imaging modality in this unique and vulnerable patient population and contribute to the existing literature on its application in specialized centers with experienced teams of radiologists, imaging technicians, and anesthesiologists.

## Materials and methods

Pediatric patients with achondroplasia referred to a quaternary pediatric neurosurgery center for neurosurgical evaluation between the years 2007 and 2023 were identified using SlicerDicer, an EMR-based cohort identification tool. Data were cross-referenced to identify patients diagnosed with achondroplasia who underwent flexion–extension cervical spine MRI to assess for possible foramen magnum stenosis during this period.

All patients referred to our institution underwent a standardized clinical evaluation based on the American Academy of Pediatrics guidelines [11]. This included a detailed history assessing feeding difficulties, choking, apnea, cyanosis, limb movement asymmetry, and tone abnormalities, with polysomnography performed when indicated. Imaging was performed in cases with findings suggestive of craniocervical stenosis; asymptomatic patients were not subjected to flexion–extension MRI. Additionally, some patients referred to our center had undergone imaging at an outside institution for other indications, such as hydrocephalus or trauma, and these scans were included in our analysis if relevant.

Patients’ medical charts were reviewed for demographic, clinical, and imaging data, including neurosurgical consultations, MRI studies, and anesthesia evaluation. Specific data points collected include the patient’s age at the time of flexion–extension MRI, gender, neurological examination findings before and after imaging, type of anesthesia (e.g., general anesthesia, sedation), MRI findings (e.g., foramen magnum stenosis and CSF flow compromise), details of any surgical interventions, and follow-up MRI results.

All patients in our cohort underwent MRI under anesthesia, which was either general anesthesia or sedation. Imaging began with the patient in the supine position, with a small bolster placed beneath the back to achieve neutral cervical spine alignment. A standardized bolster was used to ensure consistent flexion and extension positioning across all patients. Flexion was achieved by removing the bolster from under the back and placing it beneath the occiput to facilitate a chin-tuck position. An extension was achieved by removing the bolster and placing it under the back, allowing for a natural cervical extension. The degree of flexion extension was limited by the size of the head coil used during imaging. A retrospective review of the cervical angles found a mean of 25.3 ± 14° in neutral, 10.4 ± 9° in flexion, and 43 ± 14° in extension MRI. Throughout the procedure, anesthesiologists collaborated closely with the MR technologists to promptly address any airway patency concerns. All flexion–extension MRIs were performed without direct supervision by the neurosurgical service or radiologists.

The patients were examined by a senior pediatric neurosurgeon both before and on the same day after the imaging procedure under anesthesia. Neurological examinations were documented in the patients’ medical records and included a comprehensive assessment of the cranial nerve function, motor strength, reflexes, fontanel status, and overall neurological condition. Any changes or new findings following the flexion–extension MRI studies were documented with a detailed neurological examination on the same day as the imaging study.

All MRI studies included CSF flowmetry to evaluate cerebrospinal fluid dynamics at the craniocervical junction. Radiologists’ reports were reviewed to document the presence or absence of CSF flow anterior and posterior to the spinal cord during both flexion and extension. These findings were recorded for each patient and included in the analysis of imaging results.

## Results

We identified 81 patients with achondroplasia who were referred for flexion–extension MRI by a pediatric neurosurgeon to evaluate for possible foramen magnum stenosis. The cohort comprised of 43 males (53%) and 38 females (47%). The mean age at the time of MRI was 2.03 ± 2 years, with an age ranged from 1 month to 6 years. Most patients (93%) were 5 years old or younger.

All patients underwent clinical evaluations by a board-certified pediatric neurosurgeon. Among the patients, 24 exhibited neck hyperextension, 9 had exaggerated frontal bossing, 15 demonstrated weakness, 8 experienced gait disturbances, 3 had paresthesia, 12 exhibited hyperreflexia, 9 had clonus, and 16 were diagnosed with central sleep apnea confirmed by polysomnography.

A total of 124 flexion–extension MRIs were performed under anesthesia at our referral center as part of the patients’ neurosurgical evaluations. Of these, 113 MRIs were conducted under general anesthesia using a laryngeal mask airway (LMA) for airway protection, while 11 were performed under sedation. Demographics and key findings are summarized in Table 1.

**Table 1:** Demographics and key findings

Characteristics	Value
Total patients	81
Gender	43 males (53%) and 38 females (47%)
Age (mean ± SD), range	Mean, 2.03 ± 2 years Range, 1 month–6 years
Total flexion–extension MRIs	124
Anesthesia type	113 cases of general anesthesia, 11 cases of sedation
Foramen magnum stenosis	38 cases
Foramen magnum decompression surgery	38 cases

Dynamic MRI revealed foramen magnum stenosis and cord compression in 38 patients (46%). Among these patients, 32 exhibited anterior CSF flow compromise in either the flexed or extended positions, while 37 demonstrated posterior CSF flow compromise. Following the flexion–extension MRI, all 38 patients underwent surgical intervention for foramen magnum decompression. Thirty-five patients subsequently underwent follow-up flexion–extension MRI after surgery, which demonstrated complete relief of stenosis and restored CSF flow across the foramen magnum in all cases. These follow-up imaging studies were also conducted without neurosurgical or radiological supervision and were free of adverse effects or anesthesia-related complications.

Conservative management was pursued in 29 patients who had no evidence of foramen magnum stenosis at the craniocervical junction. Five of these patients underwent follow-up flexion–extension MRI under anesthesia during subsequent evaluations. In all cases, there were no adverse outcomes, changes in neurological examination, or complications associated with the imaging process.

Additionally, 12 patients were identified as having undergone flexion–extension MRI at our referral center following foramen magnum decompression that had been performed at other institutions. Of these, only 2 patients had follow-up dynamic MRI. Two additional patients were identified who underwent flexion–extension MRI as part of their neurosurgical evaluation and work-up. One of these patients was evaluated before endoscopic third ventriculostomy (ETV) and underwent follow-up imaging after surgery. The second patient was evaluated before thoracolumbar decompression. In all reported cases, no change in neurological examination or adverse events was documented following flexion–extension MRI under anesthesia.

## Discussion

**Fig. 1:**
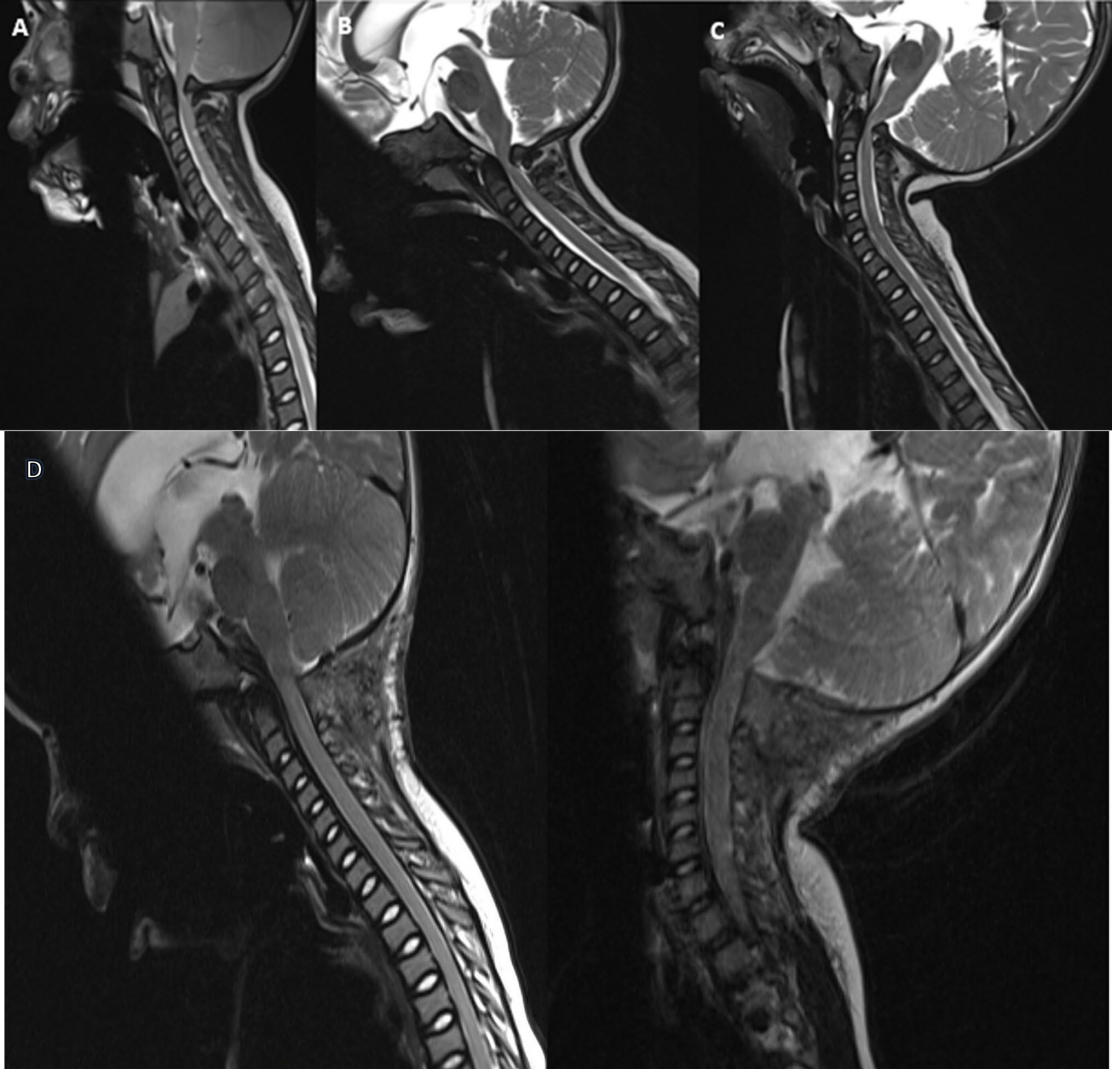
T2-weighted sagittal MRI of an 18-month-old female with achondroplasia, in neutral (**A**), flexion (**B**), and extension (**C**) positions demonstrating severe foramen magnum stenosis. **D** Post-surgical decompression results

Achondroplasia is the most common skeletal dysplasia resulting in dwarfism [2]. The abnormal development of the cranial base in patients with achondroplasia predisposes them to foramen magnum stenosis, which can lead to severe neurological complications (Fig. 1) [12]. Accurate assessment of the craniocervical junction in these patients is essential to guide medical and surgical management [6]. Dynamic MRI enables the evaluation of dynamic cord compression. For example, Mukherjee et al. [5] reported that 14% of symptomatic patients in their cohort who underwent preoperative dynamic flexion–extension CSF flow studies would not have otherwise been candidates for decompression procedures based on unremarkable neutral cervical spine MRI findings.

While dynamic MRI is widely recognized as a valuable imaging modality for this purpose, its use in pediatric patients remains limited in many institutions due to concerns about the need for anesthesia and the potential risks of neurological harm during the procedure. This study presents the largest reported dataset of pediatric patients with achondroplasia undergoing dynamic MRI to date. Our analysis included 81 patients, evaluating their neurosurgical history, pre- and post-imaging neurological examination by a senior pediatric neurosurgeon, anesthesia type, and the findings from 124 flexion–extension MRI studies performed under anesthesia without direct supervision by a neurosurgeon or radiologist. Importantly, none of the patients in our cohort experienced neurological injury, adverse events, or changes in their neurological examination as a result of the imaging procedure.

Our findings suggest that flexion–extension MRI under anesthesia did not result in observed adverse events in young children with achondroplasia, even in the absence of direct neurosurgical or radiological supervision. However, given the retrospective nature of this study, further research is needed to confirm its safety. These results support the feasibility of using this imaging modality in specialized centers with experienced anesthesiology and radiology teams.

### Limitations

The retrospective design and reliance on chart documentation may introduce bias, as the accuracy of findings depends on the completeness and reliability of recorded data. Further prospective studies are necessary to better characterize potential risks and inform clinical guidelines. Additionally, this study did not include detailed imaging findings beyond the craniocervical junction. Future research should explore broader spinal and brain anomalies to provide a more comprehensive understanding of imaging findings in achondroplasia. Moreover, the applicability of our results may be limited to institutions with extensive experience in pediatric care and may not generalize to centers with less specialized expertise. Although the absence of adverse events in our cohort is reassuring, larger multicenter studies with standardized imaging protocols are needed to validate these findings and further evaluate the safety and efficacy of this imaging modality in broader clinical contexts.

## Conclusions

In this cohort of infants and young children with achondroplasia, flexion–extension MRI under anesthesia, performed without direct supervision by a neurosurgeon or a radiologist, was not associated with adverse outcomes or neurological deficits. While these findings contribute to the understanding of the use of this imaging modality in this patient population, further research is needed to validate results and inform clinical guidelines.

## Data Availability

No datasets were generated or analysed during the current study.
